# In Vivo Assessment of Ankle Stability During Dynamic Exercises: Scoping Review

**DOI:** 10.3390/healthcare13131560

**Published:** 2025-06-30

**Authors:** Sandra Sanchez-Morilla, Pablo Cervera-Garvi, Laura Ramirez-Perez, Irene Garcia-Paya, Salvador Diaz-Miguel, Ana Belen Ortega-Avila

**Affiliations:** 1 Department of Nursing and Podiatry, Faculty of Health Sciences, Universidad de Málaga, 29071 Malaga, Spain; sandrasanchezmorilla@uma.es (S.S.-M.); pcervera@uma.es (P.C.-G.); irenegpaya@uma.es (I.G.-P.); salvadordiazm@uma.es (S.D.-M.); anaortavi@uma.es (A.B.O.-A.); 2 Department of Physiotherapy, Faculty of Health Sciences, Universidad de Málaga, 29071 Malaga, Spain; 3 IBIMA Plataforma BIONAND, 29010 Malaga, Spain

**Keywords:** ankle joint, joint instability, biomechanical phenomena, technology assessment, kinematics

## Abstract

**Background:** The ankle joint plays a key role in stabilizing the lower limb during interaction with ground reaction forces. Instability can result in pain, weakness, and impaired movement. Although assessing ankle stability is important, few studies examine existing in vivo methodologies for dynamic load assessment, limiting effective injury management. **Objective:** To identify in vivo techniques using objective measurement tools for assessing ankle stability during dynamic exercise. **Methods:** A scoping review was performed based on PRISMA-ScR criteria. Five databases—PubMed, PEDro, Embase, SPORTDiscus, and CDSR—were searched from inception to September 2024. **Results:** Out of 1678 records, 32 studies met the inclusion criteria. A total of 1142 subjects were included: 293 females (25.6%), 819 males (71.7%), and 30 unspecified (2.62%). Six categories of dynamic exercise were identified: analytical, functional, balance, stair climbing, running, and walking. The techniques used included 3D motion capture, force and pressure platforms, dynamometry, electromyography, accelerometers, pressure and speed sensors, instrumented treadmills, and inertial measurement units. **Conclusions:** The 3D motion capture systems (240 Hz) and the force platforms (1000 Hz) were most frequently used in functional tasks and walking. Combining these with multisegmented foot models appears optimal, though tool selection depends on study goals. This review enhances our understanding of ankle stability assessment.

## 1. Introduction

The ankle joint, due to its anatomical configuration, acts as a complex element of congruence within the lower limb [1]. From a functional perspective, it plays a crucial role as a stabilizer, regulating the interactions of the lower limb with ground reaction forces [2]. Since stability is the main characteristic of the ankle, any deficit in this regard may manifest through symptoms such as pain, weakness, and swelling, compromising its ability to maintain the control and balance required for movement [3]. Acute lateral ankle sprain (LAS) is a relatively common condition of the lower limb, primarily affecting the lateral ligaments of this anatomical complex [4]. Its most frequent mechanism of occurrence happens during dynamic activity, where the loads and stresses that this joint must bear increase, compromising its biomechanics [5].

A failure in the rehabilitation of the acute phase of the sprain often leads, in most cases, to the recurrence of the injury and, subsequently, to its chronicity in the patient [6]. Chronic ankle instability (CAI) is characterized by a dysfunction in both mechanical and functional stability, linked to residual symptoms from the acute lateral sprain [7]. Functional instability is associated with deficits in postural control, proprioception, and joint weakness [8]. On the other hand, mechanical instability is mainly characterized by the presence of ligamentous laxity [9]. Many patients describe it as a sensation of “giving way”, where the ankle suddenly weakens, causing a loss of control or balance [10]. The problems associated with this pathology are numerous and affect various aspects of the individual’s mobility and well-being [11]. The impairment in ankle functionality can compromise the ability to perform essential movements, leading to difficulties in carrying out everyday activities, such as walking, jumping, working, or practicing sports [12]. These impediments not only generate physical limitations but can also negatively influence the subject’s quality of life, both in their work performance and their emotional well-being, as reduced mobility impacts their autonomy and participation in recreational or social activities [13]. Furthermore, patients with chronic ankle instability present greater impairments in terms of kinesthesia and joint position sense in the affected limb compared with their healthy limb [14]; this pathology could affect not only the injured side but also the healthy limb due to the alteration of the sensorimotor system, leading to postural control issues during single-stance tasks [15], thus confirming again the influence on a patient’s daily life activities.

Considering the prevalence and impact of this pathological mechanism, its diagnosis and assessment become fundamental for appropriate therapeutic management [16]. Identifying tools for assessing ankle stability can provide categorical information to interpret the presence or absence of injury or symptoms [17]. The information in this regard is scattered in clinical practice. Typically, the primary diagnostic focus is on the patient’s medical history, physical examination, symptom identification, and the exclusion of potential fractures. In this sense, diagnostic imaging, clinical tests, and self-administered patient questionnaires have played a fundamental role [18]. However, since the ankle is characterized by complex biomechanics [19], analyzing how variables such as force or deformation influence the structures, particularly ligamentous ones, of this joint is necessary for an adequate evaluation. Although there are in vitro studies [20,21] that assess the anatomical complex of the ankle considering these aspects, there is no clear research supporting the in vivo evaluation, particularly during dynamic exercise, of ankle stability.

The objective of this study, formulated according to the PICO structure, was to identify which in vivo techniques using objective measurement instruments have been applied to evaluate ankle stability during dynamic exercise in individuals assessed for ankle instability.

## 2. Materials and Methods

### 2.1. Protocol and Registration

A scoping review was conducted following the criteria of the Preferred Reporting Items for Systematic Reviews and Meta-Analyses (PRISMA), specifically the extension for scoping reviews (PRISMA-ScR guidelines) [22]. The protocol was not registered as PROSPERO does not allow the registration of scoping reviews.

### 2.2. Eligibility Criteria

#### 2.2.1. Inclusion Criteria

In vivo observational studies were selected according to the following criteria: studies including men or women over 18 years of age, who were defined as physically active according to the WHO [23], to whom a measurement with in vivo objective techniques was applied to determine ankle instability during dynamic exercises involving ankle movement under functional conditions.

#### 2.2.2. Exclusion Criteria

Among the studies that met the inclusion criteria, the following studies were excluded: studies that assessed surgical techniques, studies that only applied an external element (plantar orthoses, footwear) in the assessment of dynamic exercise, and studies that exclusively included clinical tests, questionnaires, or imaging tests for the assessment of ankle instability.

### 2.3. Information Sources and Search

Five databases were searched from inception to September 2024: PubMed, PEDro, Embase, SPORTDiscus, and the Cochrane Database of Systematic Reviews. The keywords “load”, “ankle”, “ankle instability”, “foot”, “biomechanics”, “kinetic”, “kinematics”, “motor control”, “force”, and “stability” were searched for using the Boolean operators AND and OR. The search strategy followed in the five databases is described in Appendix A.

### 2.4. Selection of Sources of Evidence

The articles retrieved from the databases were imported into Mendeley. These were then included in Rayyan, Rayyan Systems Inc., Cambridge, MA, USA (https://www.rayyan.ai/ [accessed on 5 September 2024]). Duplicates were identified and removed. The remaining references were screened by title and abstract by two reviewers. Full-text articles were retrieved when the initial review of the title or abstract suggested that the study was eligible or when there was insufficient information in the title or abstract to assess study eligibility. Full-text articles were independently assessed for eligibility by two review authors (SSM, LRP). Disagreements about study eligibility were resolved by discussion or adjudication by two other review authors (ABOA, PCG).

### 2.5. Data Items

The following data were extracted from all the full-text articles and entered into a standardized Microsoft Word document: the authors and the year of publication, the study population, the sample size, the type of dynamic exercise evaluated, and the evaluation methodology.

### 2.6. Critical Appraisal of Individual Sources of Evidence

Following current guidelines for conducting scoping reviews [24,25], it was not necessary to include an analysis of the methodological quality. Furthermore, the lack of a standardized tool for the methodological assessment of the studies included in this review makes such an analysis difficult. In this context, this review focuses on identifying ankle stability assessment methodologies, rather than on the results obtained.

## 3. Results

### 3.1. Selection of Sources of Evidence

**Figure 1:**
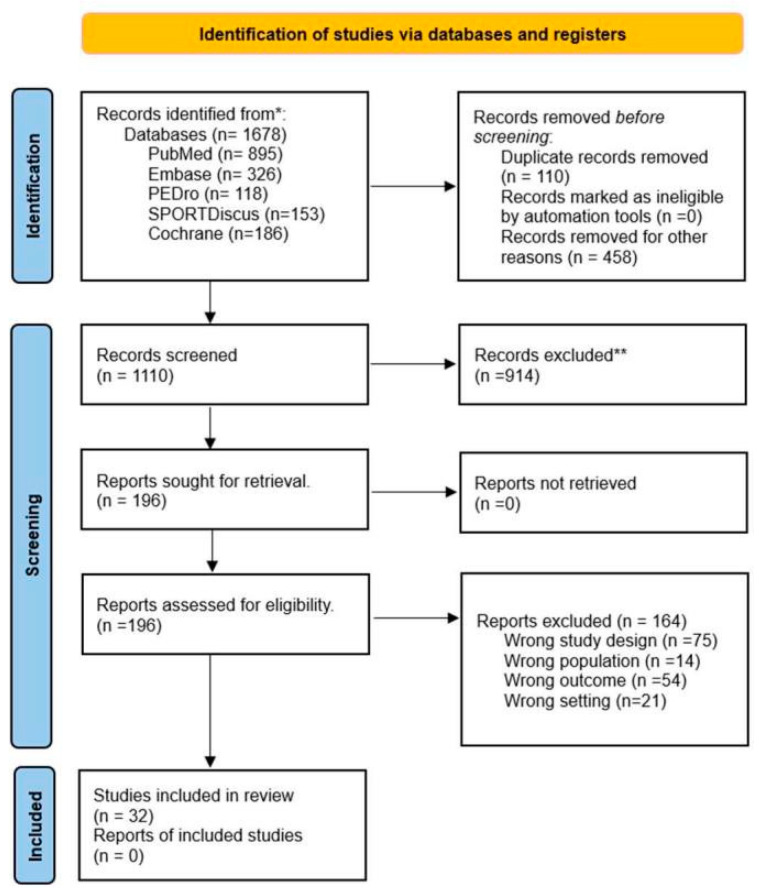
PRISMA flow diagram [26]. * Records identified in each database. ** Records excluded by Title and Abstract.

A total of 1678 records were identified in PubMed, PEDro, Embase, SPORTDiscus, and the Cochrane Database of Systematic Reviews. After screening and removing duplicates, 1110 references remained. Of these, 196 were considered potentially eligible after reviewing the titles and abstracts, and the full texts of all were retrieved. After assessing the eligibility criteria, 32 studies were finally included. Figure 1 shows the PRISMA flow diagram.

### 3.2. Characteristics of Sources of Evidence

In total, 1142 subjects were included in the studies analyzed. Of these, 293 were female (25.6%) and 819 male (71.7%). One article [27] included 30 participants without specifying their gender (2.62%).

Regarding dynamic exercises, six categories were highlighted: analytical movements [28,29,30], functional movements [31,32,33,34,35,36,37,38,39,40], balance exercises [41], stair exercises [27,42,43], running [28,44,45,46,47,48,49], and walking [50,51,52,53,54,55,56,57,58]. The assessment methodologies included two- and three- dimensional motion capture systems, pressure platforms, force platforms, hand-held dynamometers, electromyography, acceleration sensors, instrumented treadmills, speed sensors, electromagnetic sensor motion capture systems, isokinetic dynamometers, inertial measurement units, and pressure sensors.

### 3.3. Synthesis of Results

**Figure 2:**
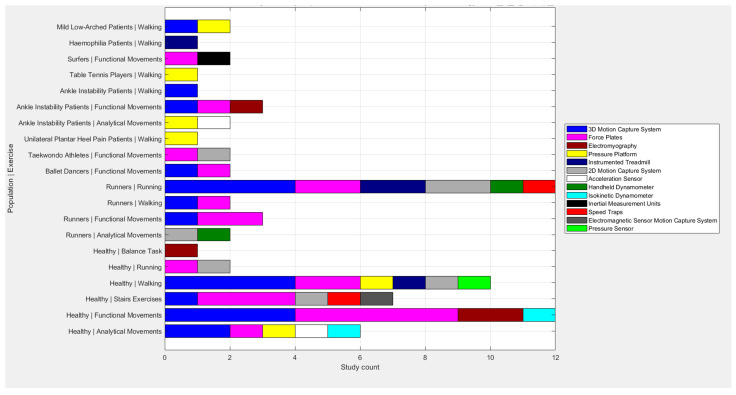
Distribution of tools per exercise and pathology.

Table 1 shows the assessment methodology in relation to the dynamic exercise carried out. Furthermore, Figure 2 graphically represents the distribution of the tools and exercises used for each pathology.

## 4. Discussion

The aim of this study was to determine the techniques with objective measurement instruments applied in vivo to assess ankle stability during dynamic exercise. The main results identified thirteen assessment methodologies, distributed into six exercise categories. The combination of 3D motion capture systems with multisegmental foot and ankle models, along with force and pressure platforms, appears to be the most suitable for assessing ankle stability during dynamic exercise.

### 4.1. Three-Dimensional Motion Capture Systems and Force-Pressure Platforms

Three-dimensional motion capture systems with markers are considered the standard for kinematic measurements [59]. Kishor Das et al. [60] highlighted their accuracy in detecting small differences in lower limb movements under various conditions. Gao Piming et al. [61] emphasized their superiority in analyzing dynamic ankle stability and understanding the mechanisms of the sprain and chronic instability. On the other hand, force-pressure platforms are widely used to assess ankle stability and functionality in kinetic terms [62]. Delahunt E et al. [63] applied force platforms to study load distribution and possible joint compensations in the lower limb. In contrast, authors such as Mckeon Po et al. [64] have pointed out that, although useful for identifying acute sprain risks, these tools are not able to adequately detect deficits related to chronic instability. However, the studies by Kyung et al. [55], Aali et al. [50], Farinelli et al. [53], Tajima et al. [56], Hashish et al. [47], and Moudy et al. [43] based this combination of assessment methodologies on their analysis of the lower limb in dynamic exercise, differing in their approaches and objectives, highlighting the complexity of capturing dynamic foot and ankle movements.

Kyung et al. used a 120 Hz 3D system together with a 50 Hz pressure platform to assess gait with the DuPont foot model [65,66] and studied intersegmental motion across different planes of movement [67,68]. Although the authors indicated that this model was detailed and had demonstrated good reliability, it was insufficient to detect small kinematic changes, showing a lack of sensitivity. They suggested the inclusion of refinements in marker-based systems combined with imaging techniques for more precise measurements. In contrast, Aali et al. used a marker placement system determined by Vicon Clinical Manager [69,70] with a 120 Hz 3D system and a 1200 Hz force platform, focusing on ground reaction forces and joint moments during walking. These authors did not report any lack of sensitivity in their results when applying this model.

In this regard, Farinelli et al. [53], using a similar configuration but with a 960 Hz platform, compared several multisegment foot models, such as the Heidelberg [71] and Oxford models [72], highlighting their ability to capture intrinsic foot and ankle movements that are often clinically significant. They critiqued the complexity of the marker placement required by these models, which can limit their practical use in gait analysis. Farinelli introduced the Distal Shank method [73] as a simplified alternative that can capture comprehensive foot power and work production more effectively, indicating that it is more accessible for clinical settings, especially in pathological cases or with children, where precise marker placement is difficult.

Tajima et al. employed the Vicon Plug-in-Gait model with a 200 Hz 3D system combined with a 1000 Hz force platform for running, without reporting any limitations in the model. However, these authors recognized the limitations of their trial sample size, noting that although three trials were analyzed, the literature suggests at least eight trials are necessary for stable values [74]. They justify their choice by referencing the reliability of kinematic and kinetic data, even with a reduced number of trials [75], though they acknowledge the potential influence of fatigue on the results. Moudy et al. also used the Plug-In-Gait body marker set, without reporting any limitations in this marker colocation system. They employed landmark registration for a waveform analysis, which increased the predictive power for performance indicators, though variability in the between-limb influence presented some challenges. Hashish used a dorsal tracking plate with skin-mounted markers affixed to the pelvic, thigh, shank, and foot segments bilaterally. The limitations noted were due to the short running path, but they did not report any inconvenience with the marker’s placement.

Edo et al. [30] and Tavakoli et al. [57] used 3D motion capture systems to assess foot and ankle kinematics. Edo et al. positioned markers on the lower limb using the Vicon Motion System. The validity and reproducibility of the method used to quantify kinematic coupling behavior were confirmed by previous studies [76]. On the other hand, Tavakoli et al. used a 3D motion capture system with reflective markers according to a single-segment model. As a limitation, they indicated that the use of a single-segment model did not specifically isolate ankle or rearfoot kinematics. However, they noted that making ground contact is a functional task for the whole foot and, initially, this model was considered appropriate. Finally, they specified that using a multisegment foot model would certainly further illuminate the kinematic events of the foot pre- and post-ground contact. Similar limitations were found in studies, such as those by Mattiussi et al. [37].

When comparing these approaches, it becomes evident that some models are more detailed in segmental foot analyses, which have been proven to be valid and reproducible by previous studies, but require precise marker placement and sophisticated setups, which may not be feasible in all settings. Farinelli’s method stands out for its clinical applicability, providing a balance between complexity and practical utility. These studies highlight the importance of the experimental setup, including the number of trials or inter-limb variability.

On the other hand, some authors, such as Balaji et al. [51] and Chow et al. [52], used pressure platforms at different frequencies to assess gait. Chow et al. measured the arch index, plantar load distribution, center of gravity, balance, and toe angle. Through static and dynamic plantar pressure analyses, they effectively assessed the load distribution in specific regions and the participants’ center of gravity balance, as well as transitions between these states. Finally, to counteract the limitations of their study, they suggested using electromyography to analyze the static and dynamic signals of the dominant leg during habitual movements and to explore the correlation between plantar load distribution, the center of gravity, and lower limb strength. Other authors, such as Malisoux et al. [36], who exclusively used force platforms to assess functional movements of the lower limb, similarly pointed out the limitations of the analysis in terms of force measurement and noted motion analysis systems or electromyography could have provided more information about biomechanical adaptations.

### 4.2. Electromyography

Electromyography allows for the analysis of muscle activation patterns, coordination, and fatigue, which are crucial for ankle stability [77]. Understanding this is essential, as altered or delayed muscle activation can increase the risk of sprains [78]. Authors such as Kessler et al. [34] and Son et al. [40] have used it in combination with other tools in their studies to assess functional exercises. However, although this technique is valuable, Qin P et al. [79] emphasize that studies using this tool exclusively on the lower limb are limited, mainly due to the influence of gravity, and recommend combining it with tools that measure kinetics and kinematics. Despite this, authors like Avila de Oliveira et al. [41] have exclusively used electromyography to evaluate balance exercises in the lower limb.

### 4.3. Instrumented Treadmills

Instrumented treadmills have recently been used due to their ability to analyze various functional aspects of the lower limb [80,81]. These treadmills allow for speed and incline adjustments, simulating diverse dynamic conditions [82]. Authors such as Coifman et al. [44] combined a 3D system with an instrumented treadmill featuring a 240 Hz sampling rate to assess running. However, other authors, such as Futrell et al. [45] and Hmida et al. [54], noted that despite this instrument’s ability to identify various parameters, such as specific gait phases, stride length and time, cadence, and pressure distribution, the constant speed used to standardize the gait during data collection may have reduced its external validity.

### 4.4. Handheld Dynamometer

The handheld dynamometer is a portable and accessible device in clinical practice that is used to assess ankle strength in different positions, making it useful for quantifying strength differences between both sides of the body [83]. However, according to Spink MJ et al. [84], its accuracy and reliability may be affected by variations between examiners, and it only measures isometric strength [85]. In contrast, the isokinetic dynamometer offers a more advanced assessment of muscle strength, allowing for the measurement of concentric and eccentric contractions throughout the movement, which improves its accuracy in evaluating fatigue, endurance, and strength imbalances [86]. Komatsu et al. [31] have used this dynamometer alongside 3D systems and force platforms to assess both functional and analytical movements.

### 4.5. Inertial Sensors

Another way to measure ankle stability is through sensors. Acceleration sensors, which measure forces along the X, Y, and Z axes [87], allow for the evaluation of the response to sudden changes in direction or speed. They have been used at frequencies of 50–1000 Hz alongside other tools by authors such as Baczkowicz et al. [29] to assess analytical movements. Pressure sensors, used by Yamamoto et al. [58] at frequencies of 200 Hz, estimate force and balance, especially when analyzing gait [88]. However, these authors pointed out that plantar pressure measurement systems are limited in that they only measure force perpendicular to the sensor surface. Therefore, other relevant forces, including shear force, cannot be measured. This system is not useful when the influence of other forces might be greater, such as in dynamic exercises, like turning or stop-and-go motions. Lastly, velocity sensors have been used by Petersen et al. [48] and DiLiberto FE et al. [42] to evaluate joint performance during stair exercises, combining them with 3D systems, force platforms, and motion capture using electromagnetic sensors, which allow for the assessment of ankle rotational movements with high sensitivity and angular precision, while being resistant to visual occlusion.

According to Franz Am et al. [89], this latest system offers advantages over 2D and 3D systems, such as position and orientation tracking with minimal processing, the detection of six degrees of freedom without requiring a direct line of sight, and simple digitization, as well as the ability to accurately capture rotational movements [90]. However, Clark R et al. [91] point out that its main limitation is its susceptibility to the distance between the transmitter and receiver and interference from elements that distort the electromagnetic field, such as metals or electrical devices. Although it provides detailed kinematic data, it does not collect kinetic data, which requires its combination with force or pressure platforms [92].

The latest tools identified are inertial measurement units, applied by Lundgren et al. [35] at frequencies of 120 Hz, combined with force platforms to evaluate functional movements. These units measure linear acceleration, angular velocity, and orientation in three dimensions [93], capturing movements with six degrees of freedom and providing real-time data on stability and balance [94]. Their cost-effectiveness makes them very useful; however, they present limitations, such as position drift due to signal noise. To correct this, authors such as Roger N et al. [95] have proposed the fusion of magnetometers and GPS, along with techniques such as extended Kalman filters.

### 4.6. Strengths and Limitations

The main limitation of this study stems from the variety and heterogeneity of the terms used; although this has been minimized by an extensive search including broad terms, some studies may not have been identified.

However, it has important strengths, most notably the methodology used, being the first study to determine and review techniques with objective measurement instruments applied in vivo to assess ankle stability during dynamic exercise. It provides information on current evaluative trends and the exercises in which these tools are used.

### 4.7. Clinical Implications

The findings of this review provide valuable guidance for clinicians in selecting the most appropriate tools for the dynamic assessment of chronic ankle instability (CAI). By highlighting the strengths and limitations of current methodologies, the review supports evidence-based decision-making in clinical settings. Integrating these methodologies, particularly when guided by the clinical context, may enhance the accuracy of diagnosis and the effectiveness of rehabilitation strategies. This comprehensive and context-driven approach could contribute to more personalized treatment plans and improved patient outcomes.

## 5. Conclusions

Thirteen objective measurement tools have been identified and applied in vivo across six categories of dynamic exercises to assess ankle stability, with 240 Hz 3D motion capture systems and 1000 Hz force platforms being the most used for kinematic and kinetic measurements. Functional exercises and walking are the primary activities for evaluation. The preferred method combines 3D motion capture with multisegmental foot models, such as force and pressure platforms, for comprehensive assessment, although the tool of choice varies based on the exercise type, resources, and scope of application.

Three-dimensional systems effectively evaluate multiplanar ankle movement but require a complex setup and model selection, while simpler 2D systems lack full spatial analyses. Force and pressure platforms offer accurate kinetic data, ideally paired with motion capture or electromyography to monitor muscle activation and fatigue. Instrumented treadmills enable controlled simulations but have limited external applicability. Electromagnetic sensors provide precise 3D rotation without visual obstruction, and inertial measurement units afford real-time stability data at a lower cost, though they suffer from signal noise. Isokinetic dynamometers assess muscle strength in both contraction phases, and velocity, acceleration, and pressure sensors contribute crucial data on movement dynamics. The integrated use of these tools supports effective injury prevention and rehabilitation.

## Figures and Tables

**Table 1:** The evaluation methodology in relation to the dynamic exercises.

References	Population	Sample Size	Type of Exercise	Evaluation Methodologies
Aali et al. (2021) [50]	Professional runners Age: 25.2 ± 1.2 Male: 13 (100%)	N = 13	Walking (aisle of 6 m): 1. Normal walking. 2. Walking with heel-first strike pattern (three attempts per each condition).	Three-dimensional motion capture system (120 Hz): Retroreflective markers placed on first and fifth metatarsals, calcaneus, medial and lateral malleoli, lateral knee joint line, lateral epicondyle of femur, anterior iliac spine, lateral surface of shank and thigh. Force plates (1200 Hz): Located in the middle of the aisle.
Abran et al. (2023) [28]	RF and NRF runners Age: 25.5 ± 4.8 Male: 80 (100%)	N = 80	Analytical movements: 1. Plantar flexion. 2. Dorsiflexion. 3. Ankle inversion. 4. Ankle eversion. 5. Flexion of first toe. 6. Flexion of the second to fifth toe. (Three trials per task.) Running: 1. Rearfoot strike. 2. Non-rearfoot strike.	Hand-held dynamometer: Fixed to a bar. Two-dimensional motion capture system (240 Hz): Reflective markers placed on the first metatarsal, lateral malleoli, lateral knee joint line, and lateral epicondyle of the femur.
Avila de Oliveira et al. (2022) [41]	University students Age: 23.27 ± 5.01 Male: 12 (50%)	N = 24	Balance task: Keeping the position while a load pulls the trunk backward. The test is performed under 8 conditions with varying foot orientations (0°–15°–30°) and loads (5–10%).	Electromyography (20–450 Hz): Wireless surface electrodes located on the medial gastrocnemius, lateral gastrocnemius, and soleus.
Baczkowicz et al. (2017) [29]	Healthy controls and chronic ankle instability patients Age: 22 ± 1.65 Male: 63 (100%)	N = 63	Analytical movements: 1. Full plantar flexion. 2. Full dorsiflexion. 3. Re-plantar flexion. (Four trials per task performed by 82 bpm.)	Acceleration sensor (50–1000 Hz): Located on the lateral malleoli. Pressure platform: The affected limb is located in the center of the platform.
Balaji et al. (2024) [51]	Patients with unilateral plantar heel pain Age: 41.11 Males: 5 (29.4%)	N = 17	Walking: Normal, straight walking.	Pressure platform: Located on the position where the second step of the patient is supposed to take place.
Chow et al. (2024) [52]	Elite and amateur table tennis players Age: 20.05 ± 0.8 Male: 147 (100%)	N = 147	Walking: Three rounds of back-and-forth normal walking.	Pressure platform: Optical plantar pressure analyzer with a sampling frequency of 15 Hz.
Coifman et al. (2024) [44]	Healthy runners Age: 24.57 ± 4.27 Male: 8 (53.3%)	N = 15	Running: Run at 3 m/s with added mass placed bilaterally as follows: 1. Foot. 2. Shank. 3. Thigh.	Three-dimensional motion capture system (240 Hz): Forty-one reflective markers with a location based on the calibrated anatomical system technique models. Instrumented treadmill (240 Hz): To record ground reaction force data.
DiLiberto et al. (2018) [42]	Healthy participants Age: 31.3 ± 4.9 Male: 6 (50%)	N = 12	Stairs exercises: Walking at 1.1 m/s plus step ascension: 1. Step of 17 cm. 2. Step of 34 cm.	Force plate (100 Hz): To collect ground reaction force data. Speed traps: Control speed at 1.1 m/s. Electromagnetic sensor motion capture system (100 Hz): Markers attached on the first, third, and fifth metatarsals, middle cuneiform, calcaneus, and tibia.
Edo et al. (2018) [30]	Young and elderly Age: 26.4 ± 3.7 73.5 ± 3.3 Male: 32 (59.3%)	N = 54	Analytical movements: Pronation and supination (six repetitions per each movement).	Three-dimensional motion capture system (240 Hz): Markers located in the fibular head, medial tibial condyle, medial and lateral malleoli, posterior, medial, and lateral surface of the heel, first and fifth metatarsals.
Erman et al. (2021) [32]	Recreational athletes Age: 24 ± 3.1 Male: 20 (100%)	N = 20	Functional movement: Body weight squat at 45 bpm.	Force plates (2000 Hz): Two plates with four load cells and single camera: evaluation with markers in the acromion, iliac spine, lateral femoral epicondyle, lateral malleolus, and first metatarsal.
Farinelli et al. (2019) [53]	Healthy subjects Age: 26 ± 2 Male: 9 (50%)	N = 18	Walking: 1. Barefoot slow, normal, and fast. 2. Shod at natural velocity.	Three-dimensional motion capture system (120 Hz): Retroreflective markers located in the medial and lateral malleoli, medial and lateral femoral epicondyles, and first and fifth metatarsals. Force plates (960 Hz): To collect ground reaction force data.
Futrell et al. (2021) [45]	Recreational runners Age: 29.7 ± 5.65 Male: 9 (27.3%)	N = 33	Running: 1. Normal speed. 2. Exerted run.	Three-dimensional motion capture system (250 Hz): Seventy retroreflective markers located on the head, trunk, upper and lower extremities according to gait analysis guides. Instrumented treadmill: To record ground reaction force data.
Garcia-Pinillos et al. (2019) [46]	Endurance runners Age: 30.9 ± 11.7 Male: 16 (88.9%)	N = 18	Running: 1. Ten runs of 400 m with 90–120” of rest. 2. Forty runs of 100 m with 25–30” of rest.	Two-dimensional motion capture system (240 Hz): Five retroreflective markers placed on the right fifth metatarsal, lateral malleolus, lateral femoral epicondyle, greater trochanter, and acromion.
Hashish et al. (2018) [47]	Recreational runners Age: 25.2 ± 2.9 Male: 6 (30%)	N = 18	Running: Performing 4–6 successful over-ground shod and barefoot running trials at 15 km/h.	Three-dimensional motion capture system (250 Hz): Markers bilaterally fixed to pelvic, thigh, shank, and foot. Force plates (3000 Hz): To record ground reaction force data.
Hmida et al. (2022) [54]	Hemophilia patients and healthy subjects Age: 37.5 ± 10.5 Male: 80 (100%)	N = 80	Walking: Normal walking at 3 km/h.	Instrumented treadmill (120 Hz): To determine the pressure distribution and ground reaction force.
Huang et al. (2019) [33]	Healthy subjects Age: not specified Male: 16 (100%)	N = 16	Functional movements: Lunge at natural speed and fast (6 attempts per each task).	Three-dimensional motion capture system (250 Hz): Sixteen markers located in the anterior and posterior-superior iliac spine, heel, toe, and lateral thigh, knee, tibia, and malleolus. Force plates (3000 Hz): To record ground reaction force data.
Kessler et al. (2020) [34]	Healthy subjects Age: not specified Male: 15 (68.2%)	N = 22	Functional movements: Three single-leg hopping tasks at slow (2 Hz), intermediate (2.3 Hz), and fast (2.6 Hz) paces.	Force plates (1125–5000 Hz): To record ground reaction force data. Electromyography: Fine-wire electrodes at abductor hallucis, flexor digitorum brevis, medial gastrocnemius, soleus, and tibialis anterior. Three-dimensional motion capture system (125–500 Hz): Twenty-four retroreflective markers located on the foot, malleolus, and femoral epicondyles. In addition, rigid marker clusters on the lateral surface of the shank.
Komatsu et al. (2024) [31]	Healthy subjects Age: 23.6 ± 1.6 Male: 13 (48.1%)	N = 27	Functional movements: 1. Forward landing. 2. Medial landing. Analytical movements at maximum isokinetic inversion.	Three-dimensional motion capture system (250 Hz): Retroreflective markers located in the 7th cervical vertebrae, 10th thoracic vertebrae, jugular notch, xiphoid process, anterolateral and posterolateral sides of the head, acromioclavicular joint, lateral epicondyles, medial and lateral sides of the wrists, 2nd metacarpal, anterior and posterior-superior iliac spine, lateral surface of the shank and the thigh, lateral femoral condyles, calcanei, lateral malleolus, and 2nd metatarsal. Force plates (1000 Hz): To record ground reaction force. Isokinetic dynamometer: Fixed to the foot, with the ankle plantar flexed at 10°, and knee flexed from 30° to 45°.
Kyung et al. (2022) [55]	Mildly low-arched patients and control group Age: 23.5 ± 1.15 Male: 30 (100%)	N = 30	Walking: Normal walking wearing a 20 kg backpack.	Three-dimensional motion capture system (120 Hz): Fifteen markers placed on the lateral and medial joint line of the knee, tibia, medial and lateral malleoli, hindfoot segment, heel, navicular, cuboid, first and fifth metatarsal, and hallux. Pressure platform: Four sensors/cm^2^ operating at 50 Hz.
Lundgren et al. (2015) [35]	Surfing athletes Age: 24 ± 6.9 Male: 11 (100%)	N = 11	Functional movements: Landing in a backhand and a forehand position (five trials of each task). In addition, two gymnastics landing tasks from a mini-trampoline to a soft mat.	Inertial measurement units (120 Hz): Sensors bilaterally located at the mid-foot, mid-tibial plateau, T8, and S2. Force plates (600 Hz): To record ground reaction force data.
Malisoux et al. (2017) [36]	Physically active and healthy subjects Age: 26.8 ± 5.7 Male: 41 (100%)	N = 41	Functional movements: 1. Single ankle jump. 2. Two consecutive maximal counter-movement jumps (each task was performed on four different sport floorings).	Force plates (1000 Hz): To record ground reaction force data.
Mattiussi et al. (2024) [37]	Professional ballet dancers Age: 25.4 ± 4.3 Male: 14 (51.9%)	N = 27	Functional movements: 1. Unilateral squat. 2. Unilateral standing plantarflexion. 3. Unilateral seat plantarflexion. 4. Weight-bearing lunge. 5. Counter-movement jumps.	Force plates (1000 Hz): To record ground reaction force data. Three-dimensional motion capture system (200 Hz): Retroreflective markers located in greater trochanter, medial and lateral joint lines of the knee, medial and lateral malleoli, calcaneus, navicular, first and fifth metatarsal. In addition, a four-marker cluster on the lateral side of the shank.
Moudy et al. (2020) [43]	Healthy subjects Age: 34 ± 6.5 Male: 22 (100%)	N = 22	Stairs exercises: Normal walking and stepping down from a 14 cm step using the toe and using the heel.	Three-dimensional motion capture system (200 Hz): Full-body marker set according to Plug-In-Gait model. Force plates (1000 Hz): To record ground reaction force data.
Petersen et al. (2014) [48]	Recreational runners Age: 25 ± 6 Male: 16 (48.5%)	N = 33	Running: Normal running at 8 km/h, 12 km/h, and 16 km/h.	Three-dimensional motion capture system (240 Hz): Thirty-six retroreflective markers located on the pelvis, thighs, shanks, and feet. Force plates (960 Hz): To record ground reaction force data. Speed traps: Place 3 m apart before and after the force plate.
Powell et al. (2016) [38]	Recreational runners Age: 20.1 ± 2.4 Male: 0 (0%)	N = 20	Functional movements: Barefoot step-off landing from a box of 30 cm (Five successful repetitions).	Three-dimensional motion capture system (240 Hz): Thirty-six retroreflective markers located in the first and fifth metatarsal, medial and lateral malleoli, medial and lateral femoral epicondyles, greater trochanters, anterior and posterior-superior iliac spines. In addition, four-marker clusters on the pelvis, thigh, and shank. Force plates (1200 Hz): To record ground reaction force data.
Rutkowska-Kucharska et al. (2017) [27]	University students Age: 22.6 ± 0.65 Male: not specified.	N = 30	Stairs exercise: Twelve barefoot steps at 123 bpm.	Two-dimensional motion capture system (120 Hz): Reflective markers located on 7th cervical vertebrae, acromion, anterior superior iliac spine, sacrum, greater trochanter, lateral femoral epicondyle, fibula head, lateral malleolus, fifth metatarsal, and calcaneus. Force plates (1000 Hz): To record ground reaction force data.
Ryu et al. (2021) [39]	Taekwondo athletes Age: 22 ± 2.4 Male: 17 (100%)	N = 17	Functional movements: unilateral jumping, kicking, and landing (Five attempts per each task).	Two-dimensional motion capture system (200 Hz): Eighteen reflective markers located on the acromion, humerus lateral epicondyle, ulnar styloid process, iliac crest, greater trochanter, femoral condyles, malleolus, heel, and 2nd phalange. Force plates (2000 Hz): to record ground reaction force data.
Son et al. (2017) [40]	Chronic ankle instability patients, ankle sprain copers, and healthy controls Age: 22.2 ± 2.13 Male: 36 (54.5%)	N = 66	Functional movements: Maximal vertical forward jump, unilateral landing, and side-cut at 90° to the contralateral side as fast as possible (ten attempts per task).	Three-dimensional motion capture system (240 Hz): Fifty-one reflective markers according to the full-body marker set. Force plates (1200 Hz): To record ground reaction force data. Electromyography (1200 Hz): Electrodes placed over the tibialis anterior, peroneus longus, gastrocnemius, vastus lateralis, hamstring, gluteus medius, and maximus.
Tajima et al. (2018) [56]	Healthy subjects Age: 21.6 ± 0.7 Male: 6 (42.9%)	N = 14	Walking: Normal and impact reduction walking at 110 steps/minute.	Three-dimensional motion capture system (200 Hz): Thirty-five reflective markers according to the Vicon Plug-in-Gait model. Force plates (1000 Hz): To record ground reaction force data.
Tavakoli et al. (2016) [57]	Functional ankle instability patients and healthy controls Age: 25.26 ± 3.95 Males: 22 (55%)	N = 40	Walking: 1. Normal walking. 2. Normal walking doing a cognitive task.	Three-dimensional motion capture system (200 Hz): Reflective markers located in the first, second, and fifth metatarsals, as well as the calcaneus, medial and lateral femur epicondyles, and medial and lateral malleoli. In addition, a four-marker cluster is attached to the lateral surface of the shank.
Wang et al. (2022) [49]	Active young adults Age: 21.9 ± 1.8 Male: 4 (28.6%)	N = 14	Walking and running: 1. Normal walking. 2. Normal running. 3. Walking with backpack weight. 4. Running with backpack weight.	Two-dimensional motion capture system (200 Hz): Thirty-one markers placed on the head, upper extremities, pelvis, lower extremities, and feet, according to the Helen Hayes model. Force plates (1000 Hz): To record ground reaction force data.
Yamamoto et al. (2020) [58]	Healthy subjects Age: 32.5 ± 7.5 Male: 50 (50%)	N = 100	Walking: Ten steps at normal velocity.	Pressure sensor (200 Hz): Ten sensors of 1 mm thickness and 12 g weight placed on the toes, forefoot, midfoot, and hindfoot.

BPM: beats per minute; RF: rearfoot, NRF: non-rearfoot, Hz: Hertz, N: sample size.

## Data Availability

The data supporting the findings of this study are not publicly available due to privacy and ethical restrictions.

## Abbreviations

The following abbreviations are used in this manuscript:

LAS	lateral ankle sprain
PRISMA	Preferred Reporting Items for Systematic Reviews and Meta-Analyses
PRISMA-ScR	PRISMA extension for scoping reviews
PICO	Participants, Intervention, Comparison, Outcome
WHO	World Health Organization
Hz	Hertz
N	sample size
RF	rearfoot
NRF	non-rearfoot
3D	three-dimensional
Vicon	Vicon Motion Systems
EMG	electromyography
2D	two-dimensional
BPM	beats per minute

## Appendix A

### Search Strategy

1	Load
2	Foot
3	Ankle
4	Stability
5	Ankle instability
6	Biomechanics
7	Kinetics
8	Kinematics
9	Motor control
10	Force
11	1 AND 2 OR 3
12	2 AND 6
13	7 OR 8 AND 5
14	9 AND 5
15	10 AND 3 AND 4
